# Dermoscopy of nipple adenoma

**DOI:** 10.1002/ccr3.3398

**Published:** 2020-10-13

**Authors:** Mahshid Sadat Ansari, Jafar Taghizadeh Fazli, Amirhooshang Ehsani

**Affiliations:** ^1^ Autoimmune Bullous Diseases Research Center Department of Dermatology Tehran University of Medical Sciences Tehran Iran; ^2^ Department of Dermatopathology Tehran University of Medical Sciences Tehran Iran

**Keywords:** breast, dermoscopy, nipple, nipple adenoma

## Abstract

Characteristic finding of nipple adenoma (NA) in dermoscopy (red dots in linear, radial, or semicircular patterns) can help in accurate clinicopathologic diagnosis of NA vs other inflammatory, benign, and especially malignant nipple lesions.

## Graphical abstract

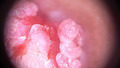

## 1 INTRODUCTION

Nipple adenoma (NA) is a benign neoplasm of lactiferous ducts. It can be misdiagnosed as malignant nipple lesions. Dermoscopy was done for a 22‐year‐old woman with histopathologically confirmed NA. Dermoscopy helps in the correct diagnosis of the lesion and in avoiding unnecessary wide excision of the lesion.

Nipple adenoma (NA) is a rare benign neoplasm of lactiferous ducts that affect middle‐aged women,^1^ but it has been reported in children as well.^2^ Florid papillomatosis or papillary adenoma of the nipple, erosive adenomatosis of the nipple, nipple duct adenoma, and florid adenomatosis of the nipple are some other names of the disease, historically.^2^ NA was named according to 2012 WHO classification of breast tumors.^3^ It presents as a palpable nipple nodule, erosive lesion, with or without discharge from the skin surface.^3^ NA may be misdiagnosed as mammary Paget's disease (MPD) clinically and intraductal carcinoma histologically.

Dermoscopy in combination with histopathologic studies can be useful in accurate diagnosis of NA.^4^ Treatment of choice is complete surgical excision to prevent local recurrence^1^, ^3^; other treatments include Mohs micrographic surgery, cryotherapy, and radiofrequency.^3^ To the best of our knowledge, dermoscopy of NA has been done in three cases to date.^3^, ^4^, ^5^

## 2 CASE PRESENTATION

**FIGURE 1:**
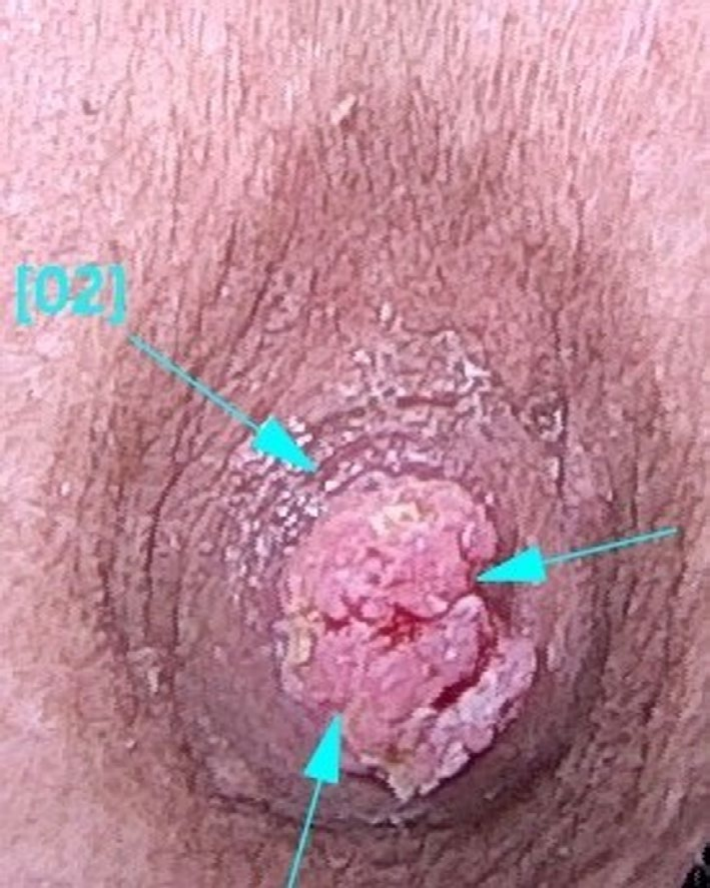
Papillomatous lesion with erosion and ulcerations on the nipple

**FIGURE 2:**
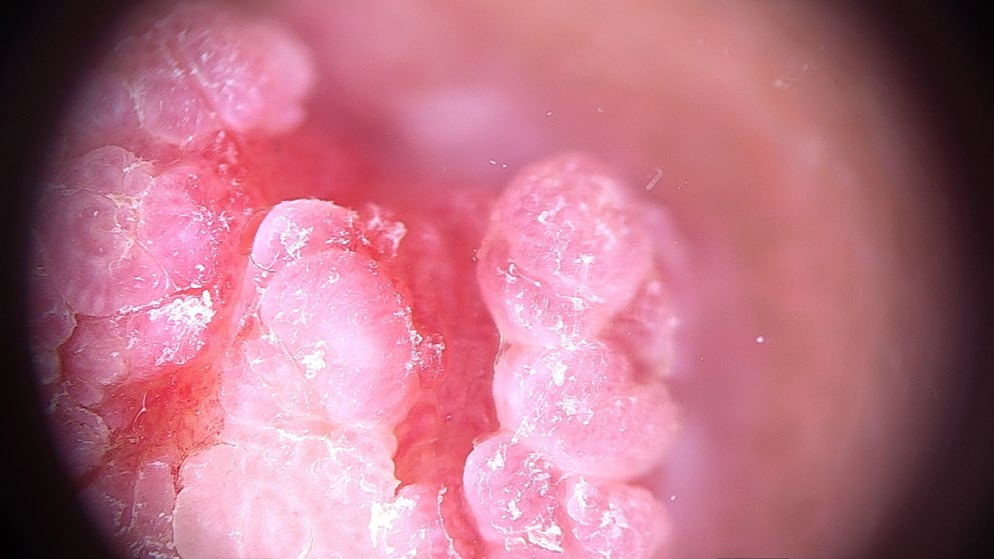
Pink‐white clods, red structureless areas, and red dots in linear, radial, or semicircular patterns

**FIGURE 3:**
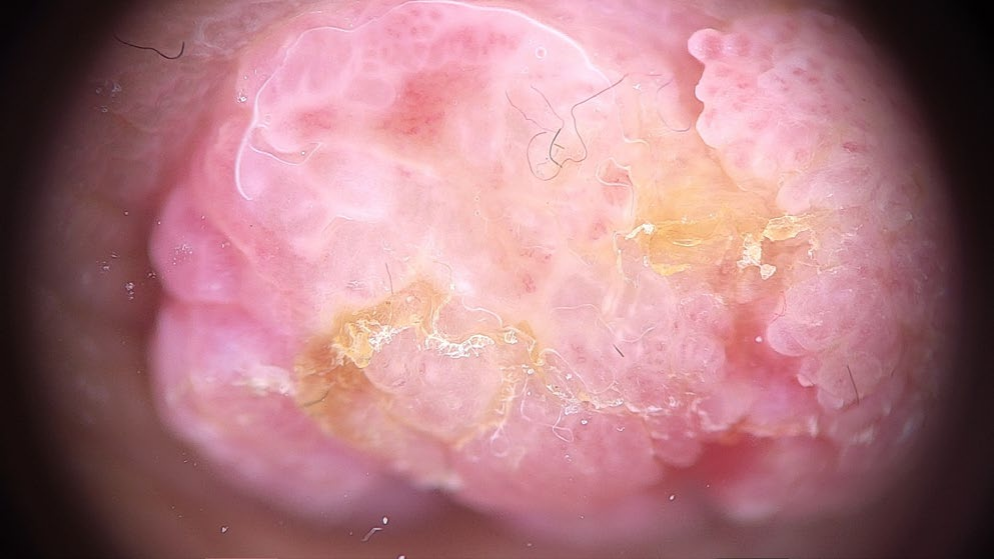
Hyperkeratotic white and yellow scale and red globules. Dermoscopic images were taken by polarized light with 20‐fold magnification, FotoFinder videodermoscope

**FIGURE 4:**
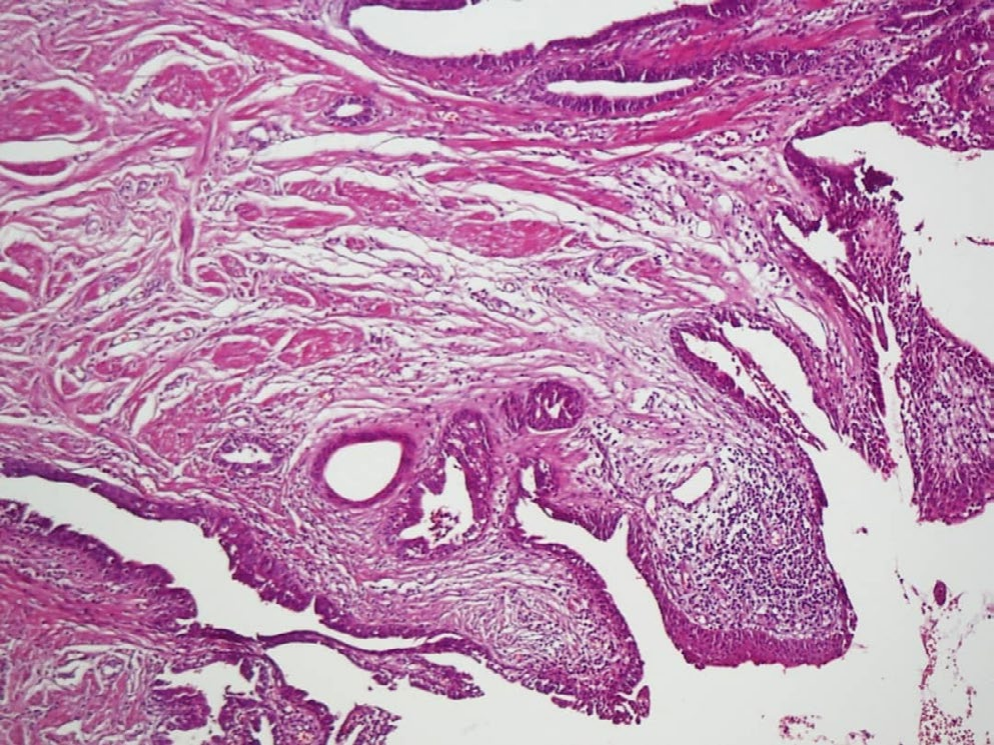
Marked papillomatous changes, non‐encapsulated well‐circumscribed adenomatous lesion in dermis, and duct‐like structures of variable sizes. Some ducts connect with the surface epithelium (H&E, ×40)

A 22‐year‐old woman referred to our hospital has presented with a papillomatous nodule on the left nipple from childhood (Figure 1). Skin biopsy with punch number 3 was done. Histopathologic study was compatible with NA. Videodermoscopy was done with FotoFinder dermoscope, Bad Birnbach, Germany. The lesion had 2 parts in videodermoscopy: red structureless areas and pink‐white clods. Red structureless areas were composed of red dots in a pink background. Pink‐white clods were composed of a white background and red dots in linear, semicircular, and radial patterns (Figure 2). Some red globules and red lines were seen in both parts as well. White and yellow hyperkeratotic scales were seen mostly on clods, and they could obscure red dots (Figure 3). The tumor was completely excised, and histopathologic study confirmed the diagnosis of NA. It showed skin tissue with well‐circumscribed, nonencapsulated tubuloglandular tumor directly connected to overlying epidermis with erosion and interspaced epidermis. Tumor was composed of ducts and tubules with papillary projections into dilated tubular spaces lined by bland‐looking epithelial cells and a backing of myoepithelial cells (Figure 4).

## 3 DISCUSSION

Takishama and colleagues have reported one case of NA dermoscopy in 2015 and found regular, small, linear cherry red structures characteristically that reflect luminal opening in histopathology, also nonspecific orange veil structures at the periphery that correspond with the remaining epidermis.^4^ Sophn et al have reported another NA dermoscopy case in 2016, with increased red serpiginous and annular structures.^3^ In 2017, Errichetti and colleagues have reported another case with sparse dotted vessels that correlate with vascularity in histopathology, on a reddish‐whitish background and whitish‐yellowish hyperkeratosis.^3^ Our case was a completely developed NA and presented all dermoscopic signs. Structureless red areas and pink‐white clods correspond to erosion and interspaced epidermis in histopathology, respectively. Red dots in linear, radial, semicircular patterns correlate with tubular and luminal openings in histopathologic studies. Red globules and red lines correspond to regular vessels of a benign tumor. The most important clinical differential diagnosis of NA is mammary Paget's disease. Dermoscopic signs include light brown diffuse pigmentation, irregularly distributed blue‐gray dots, irregular black dots, and irregular linear vessels. It seems that the dominance of red and white color and regularity are the signs that guide a dermatologist to the diagnosis of NA.^5^

Patients with NA mostly refer to dermatologists as their lesion presents on their skin. Because NA is a very rare disease, a dermatologist must consider the diagnosis in the mind. Characteristic finding of NA in dermoscopy (red dots in linear, radial, or semicircular patterns) can help in accurate clinicopathologic diagnosis of NA vs other inflammatory, benign, and especially malignant nipple lesions such as MPD.

In conclusion, dermoscopy can help dermatologist to guess the most accurate diagnosis of the lesion and it helps in preventing the patient to undergo unnecessary wide surgery, also a great psychological stress.

## CONFLICT OF INTEREST

The authors have no conflict of interest to declare.

## AUTHOR CONTRIBUTIONS

MSA: contributed to conception and design, draft of the manuscript, acquisition of data, and analysis and interpretation of data. JTF: contributed to acquisition of data, and analysis and interpretation of data. AE: contributed to revision the manuscript critically for important intellectual content and gave final approval of the version to be published.

## INFORMED CONSENT

The patient filled informed consent form to her liking. Images did not identify the patient.

## Data Availability

The authors confirm that the data supporting the findings of this study are available within the article and/or its supplementary materials.
